# Parallel Lives

**DOI:** 10.1371/journal.pbio.1000528

**Published:** 2010-10-26

**Authors:** Robin Meadows

**Affiliations:** Freelance Science Writer, Fairfield, California, United States of America

**Figure pbio-1000528-g001:**
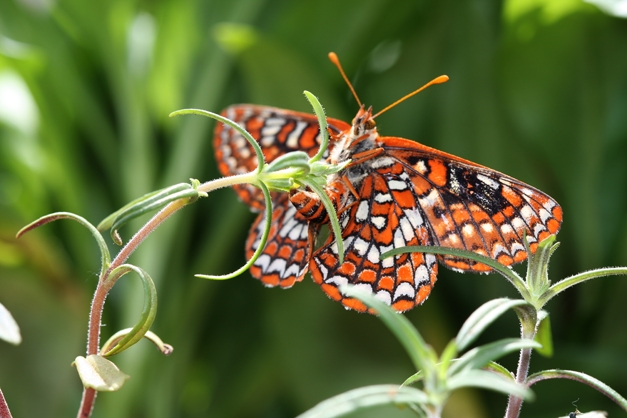
While the female above lays small clutches of eggs near the a plant's tip, females of the same species, *Euphydryas editha*, from allopatric populations that specialize on a different host plant, lay large clutches near the ground. Field studies show that “hybrids” have intermediate traits that cause them to suffer reduced fitness, revealing a form of reproductive isolation can provide a substantial barrier to gene flow at the early stages of ecological divergence and speciation. Image: Damien Caillaud, University of Texas at Austin.

[Fig pbio-1000528-g001]California's Sierra Nevada mountains are dotted with populations of checkerspot butterflies that belong to the same species but lead separate lives. The populations are differentiated by the host plants where females lay their eggs, with one type favoring *Collinsis torreyi* (blue-eyed Mary) and the other favoring *Pedicularis semibarbata* (pine lousewort). Other than that, the checkerspots are much the same. They are alike morphologically and genetically, and can also interbreed and produce hybrid offspring. So what does keep them apart? New work by Carolyn McBride and Michael Singer in this issue of *PLoS Biology* reveals it is traits that let each type get the most out of its chosen host plants, but put hybrids at a disadvantage on either plant.

Such divergence of populations, where offspring are viable but maladapted due to intermediate traits not suited to either ecological niche, is called extrinsic postzygotic isolation (EPI) and is thought to be an early stage in the formation of new species. However, there are few examples of EPI and most are in cases where strong barriers to mating already exist, leaving the extent of EPI's contribution to speciation in question. The apparent lack of mating barriers between the Sierra Nevada checkerspot populations makes this a particularly good system for teasing out the relative strength of EPI as a driver of speciation.

The traits that distinguish the butterfly populations stem from key differences in the two host plants, which grow intermingled on the western slopes of the mountains. Blue-eyed Mary is a short-lived annual with leaves that die from the bottom up, and female checkerspots adapted to this host plant lay their eggs on the nutritious new growth near the top. In contrast, pine lousewort is a perennial that stays green all summer and grows new leaves from the base, and females adapted to this host plant lay their eggs near the bottom.

To see if EPI is nudging these butterfly populations apart, McBride and Singer asked whether hybrids were intermediate for traits that distinguish the parents, including where females lay their eggs, clutch size, where larvae forage, and larval growth and survival. The researchers discovered that hybrids were somewhere in the middle for all traits tested. For instance, whereas the two parent populations prefer the host plants to which they are adapted, hybrid females readily lay their eggs on either host.

Next, the researchers asked whether these intermediate traits put hybrids at a disadvantage in the wild. Hybrids were perfectly healthy when raised in the laboratory, indicating that they had no intrinsic defects, but fared poorly under natural conditions. Field tests revealed that each intermediate behavior was maladaptive, with intermediate egg laying behaviors being the most disadvantageous. Whereas “blue-eyed Mary” females preferred plants that were still in bud, hybrids preferred blue-eyed Mary plants that were already in bloom, and laying eggs on these older plants cut offspring survival 70%. Moreover, whereas “blue-eyed Mary” females lay their eggs near plant tops and “lousewort” females lay theirs near the ground, hybrid females lay theirs at intermediate heights on lousewort, and larvae there grew roughly 50% slower than those near the ground. Measuring larval growth on lousewort entailed individually super-gluing about 30 eggs to leaves and there were 12 such clutches at each of 14 field sites, making this perhaps the most painstaking of the tremendous amount of work behind these field tests.

In addition, “blue-eyed Mary” females lay relatively small clutches of up to 20 eggs and “lousewort” females lay larger clutches of up to 100. But hybrid females lay medium-sized clutches that are at a disadvantage on both host plants: larval survival was higher for smaller clutches on blue-eyed Mary and for larger clutches on lousewort. This difference is likely due to higher rates of egg predation on lousewort.

To visualize how the distinguishing traits isolate the two butterfly populations, the researchers constructed an “adaptive surface” or fitness landscape by plotting host preference and clutch size against offspring survival. The resulting landscape neatly displays two fitness peaks separated by a maladaptive valley, with one of the parent butterflies on each peak and hybrids lying in the valley.

Ecological selection against hybrids has been demonstrated in a few species including threespine sticklebacks and Darwin's finches. However, it has been difficult to sort out the relative impact of EPI from other forms of reproductive isolation such as mating barriers and geographical separation. By showing that behavioral differences alone are enough to keep the Sierra Nevada checkerspot populations apart in the wild as well as by demonstrating that hybrids fare poorly in the wild due to intermediate traits, this work makes a compelling case that EPI can play a strong and perhaps even primary role in the onset of new species formation.

Other cases where diverging populations produce hybrids with adverse intermediate behaviors include the European blackcap, a migratory warbler that winters in southern Europe and Africa. Some of these birds migrate southeast and others southwest, and hybrids between the two choose an intermediate direction that can lead to daunting flightpaths over the Alps, the Mediterranean Sea, and the Sahara Desert. Because adaptation to new niches is often accompanied by preferences for that niche, such maladaptive intermediate behaviors may be a widespread driver of EPI amongst animals. Furthermore, most previous estimates of EPI are from laboratory studies that fail to account for real-world challenges such as predators and pathogens, raising the possibility that the impact of ecological selection has been underestimated. Taken together, these considerations suggest that EPI may be a more significant driver of speciation than previously suspected.

**McBride CS, Singer MC (2010) Field Studies Reveal Strong Postmating Isolation between Ecologically Divergent Butterfly Populations. doi:10.1371/journal.pbio.1000529**

